# Redetermination of the crystal structure of *R*_5_Si_4_ (*R* = Pr, Nd) from single-crystal X-ray diffraction data

**DOI:** 10.1107/S2056989020002789

**Published:** 2020-03-10

**Authors:** Kaori Yokota, Ryuta Watanuki, Miki Nakashima, Masatomo Uehara, Jun Gouchi, Yoshiya Uwatoko, Izuru Umehara

**Affiliations:** a Department of Physics, Yokohama National University, 79-5 Tokiwadai, Hodogaya-ku, Yokohama 240-8501, Japan; b Department of Chemistry, Yokohama National University, 79-5 Tokiwadai, Hodogaya-ku, Yokohama 240-8501, Japan; c The Institute for Solid State Physics, The University of Tokyo, 5-1-5, Kashiwanoha, Kashiwa, Chiba 277-8581, Japan

**Keywords:** crystal structure, rare-earth silicide, chiral structure, chiral magnet, single-crystal growth, X-ray diffraction

## Abstract

The crystal structures of praseodymium silicide (5/4), Pr_5_Si_4_, and neodymium silicide (5/4), Nd_5_Si_4_, were redetermined using high-quality single-crystal X-ray diffraction data. The previous structure reports of Pr_5_Si_4_ were only based on powder X-ray diffraction data [Smith *et al.* (1967 ▸). *Acta Cryst.* **22** 940–943; Yang *et al.* (2002*b* ▸). *J. Alloys Compd.* **339**, 189–194; Yang *et al.*, (2003 ▸). *J. Alloys Compd.* **263**, 146–153]. On the other hand, the structure of Nd_5_Si_4_ has been determined from powder data [neutron; Cadogan *et al.*, (2002 ▸). *J. Phys. Condens. Matter*, **14**, 7191–7200] and X-ray [Smith *et al.* (1967 ▸). *Acta Cryst.* **22** 940–943; Yang *et al.* (2002*b* ▸). *J. Alloys Compd.* **339**, 189–194; Yang *et al.*, (2003 ▸). *J. Alloys Compd.* **263**, 146–153] and single-crystal data with isotropic atomic displacement parameters [Roger *et al.*, (2006 ▸). *J. Alloys Compd.* **415**, 73–84]. In addition, the anisotropic atomic displacement parameters for all atomic sites have been determined for the first time. These compounds are confirmed to have the tetra­gonal Zr_5_Si_4_-type structure (space group: *P*4_1_2_1_2), as reported previously (Smith *et al.*, 1967 ▸). The structure is built up by distorted body-centered cubes consisting of Pr(Nd) atoms, which are linked to each other by edge-sharing to form a three-dimensional framework. This framework delimits zigzag channels in which the silicon dimers are situated.

## Abstract 2

The crystal structures of praseodymium silicide (5/4), Pr_5_Si_4_, and neodymium silicide (5/4), Nd_5_Si_4_, were redetermined at higher precision. These compounds are confirmed to have the tetra­gonal Zr_5_Si_4_-type structure (space group: *P*4_1_2_1_2). The structure is built up by distorted body-centered cubes consisting of Pr(Nd) atoms, which are linked to each other by edge-sharing to form a three-dimensional framework. Silicon dimers are located within channels in this framework.

## Chemical context   

In natural science, there are some essential concepts concerned with symmetry, among which chiral symmetry is one of the fundamentals in all fields of physics, especially magnetism in solid-state materials. A chiral magnet in solids is of great inter­est in both science and technology. These magnets have been studied for novel phenomena such as chiral magnetic soliton lattices and use in future spintronic devices such as magnetic memories and logic gates. The critical point is that the crystal-structure chirality affects the arrangement of magnetic moments in these materials. The symmetry of crystals plays an important role in the spatial arrangement of the magnetic moments. For example, the inter­metallic compound YbNi_3_Al_9_ has a trigonal ErNi_3_Al_9_-type structure in space group *R*32, a member of the Sohncke group (Gladyshevskii *et al.*, 1993 ▸). This compound exhibits a characteristic helical magnetic structure, reflecting the symmetry of the crystal (Aoki *et al.*, 2018 ▸). To study magnetism for chiral symmetry, we focused on the inter­metallic compound *R*_5_Si_4_ (*R* = Pr and Nd), which has a tetra­gonal Zr_5_Si_4_-type crystal structure in the chiral space group *P*4_1_2_1_2 (Smith *et al.*, 1967 ▸).

Roger *et al.* (2006 ▸) isolated a small single crystal of Nd_5_Si_4_ by crushing the solidified sample and collected single-crystal X-ray data. Very recently, Sato *et al.* (2018 ▸) reported the single-crystal growth and magnetic properties of Ce_5_Si_4_, which has the same crystal structure as Pr_5_Si_4_ and Nd_5_Si_4_. At present, there has only been a report of large-size single-crystal growth for *R* = Ce, and there are no reports of a large single crystal having been grown successfully for *R* = Pr or Nd. In particular, for Pr_5_Si_4_, the crystal-structure analysis is based only on powder XRD data (Yang *et al.*, 2002*a* ▸,*b* ▸,*c* ▸, 2003 ▸; Cadogan *et al.*, 2002 ▸; Smith *et al.*, 1967 ▸). It is still unknown, however, whether there is a relationship between chiral symmetry and electronic properties, including magnetic ones. In this paper, we report the details of crystallographic studies of single-crystal X-ray analysis of high-quality single-crystalline Pr_5_Si_4_ and Nd_5_Si_4_, which are expected to be candidate materials for chiral magnets.

## Structural commentary   

**Figure 1:**
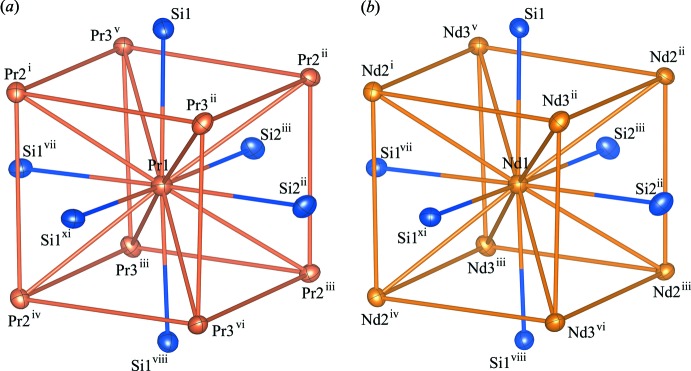
Principal units in the structure of (*a*) Pr_5_Si_4_ and (*b*) Nd_5_Si_4_, illustrated using *VESTA* (Momma & Izumi, 2011 ▸). Displacement ellipsoids are drawn at the 90% probability level. Symmetry codes: (i) −*y* + 1, −*x* + 1, −*z* + 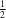; (ii) −*y* + 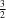, *x* + 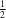, *z* + 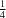; (iii) *x* + 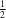, −*y* + 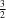, −*z* + 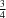; (iv) −*x* + 1, −*y* + 1, *z* + 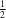; (v) −*y* + 2, −*x* + 1, −*z* + 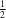; (vi) −*x* + 1, −*y* + 2, *z* + 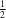; (vii) −*y* + 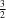, *x* − 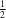, *z* + 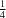; (viii) *y*, *x*, −*z* + 1; (ix) *x* − 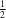, −*y* + 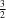, −*z* + 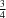.

**Figure 2:**
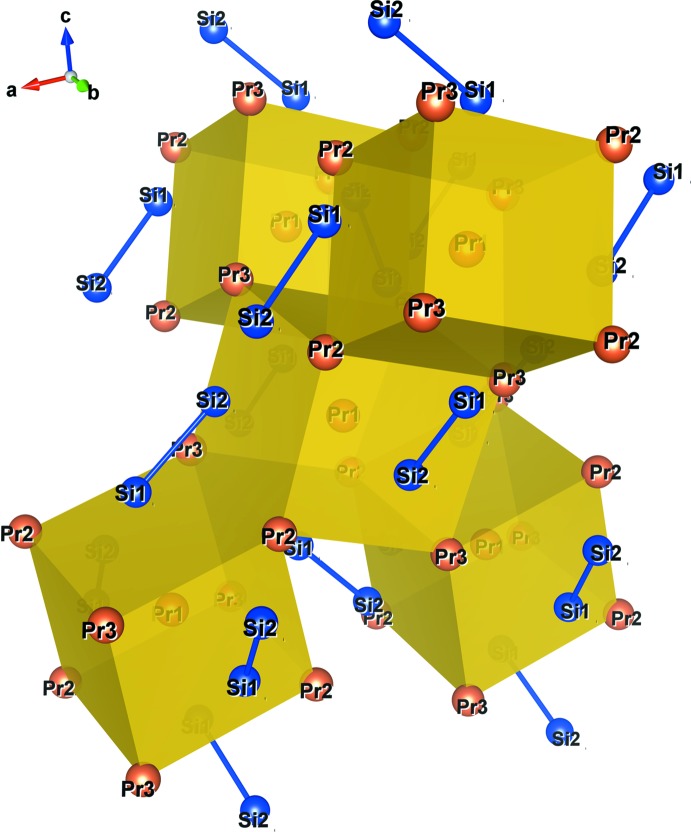
Parts of the crystal structure showing five distorted body-centered cubes sharing Pr2—Pr3 edges (polyhedral drawing). Si1 and Si2 atoms form dimers with atoms Si2 and Si1, respectively, of the adjacent unit.

**Figure 3:**
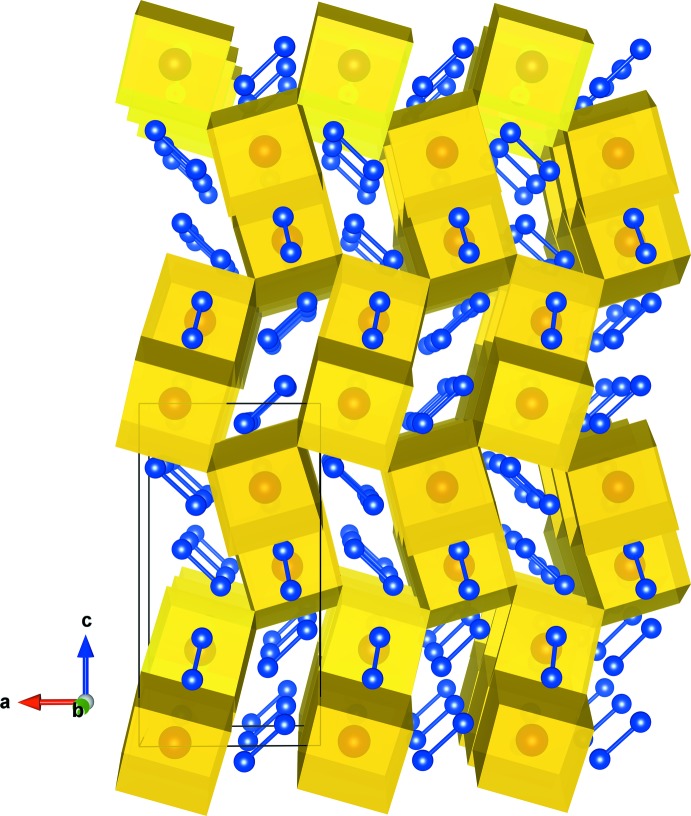
Polyhedral representation of the crystal structure of Pr_5_Si_4_ showing the Si–Si dimers situated in zigzag channels running along the [100] and [010] directions.

The crystal structures of Pr_5_Si_4_ and Nd_5_Si_4_ refined in this study are essentially the same as those determined previously, belonging to chiral space group *P*4_1_2_1_2 (No. 92) for *R* = La, Ce, and Nd (Yang *et al.*, 2002*a* ▸; Sato *et al.*, 2018 ▸). The asymmetric unit of these compounds consists of three Pr (Nd) and two Si atoms. The Pr1(Nd1) atom occupies the Wyckoff 4*a* site, and the Pr2 (Nd2), Pr3 (Nd3), Si1 and Si2 are located on the general position 8*b* sites. The principal units in the crystal structures of Pr_5_Si_4_ and Nd_5_Si_4_ are illustrated in Fig. 1 ▸, and selected bond lengths are given in Tables 1 ▸ and 2 ▸. The Pr1(Nd1) coordination environment in these compounds can be described as a distorted cube with four Pr2 (Nd2) and four Pr3 (Nd3) [Pr1—Pr2 and Pr1—Pr3 bond lengths ranging from 3.4914 (4) to 3.6423 (3) Å, Pr2—Pr3 bond lengths in the range 3.9156 (3) to 4.0074 (2) Å, Nd1—Nd2 and Nd1—Nd3 bond lengths of 3.4725 (5)–3.6265 (3) Å and Nd2—Nd3 bond lengths of 3.9094 (4)–3.9752 (2) Å]. In addition, the Pr1(Nd1)—Si bonds protruding through the distorted rectangular faces formed by two Pr2 (Nd2) and two Pr3 (Nd3) atoms have Pr1—Si bond lengths ranging from 3.0985 (13) to 3.1780 (13) Å and Nd1—Si bond lengths from 3.0744 (15) to 3.1661 (16) Å. The distorted cubes are connected through common two Pr2—Pr3 (Nd2—Nd3) edges, and Si1 (Si2) atoms form dimers with Si2 (Si1) atoms in the adjacent unit (Fig. 2 ▸). The Si1—Si2 bond length in Pr_5_Si_4_ is 2.4738 (16) Å, and that of Nd_5_Si_4_ is 2.482 (2) Å. The extended structure is shown in polyhedral representation in Fig. 3 ▸. The structure is built up by distorted body-centered cubes consisting of Pr (Nd) atoms, which are linked to each other by edge-sharing to form a three-dimensional framework. This framework delimits zigzag channels oriented along the [100] and [010] directions, in which the Si–Si dimers are situated.

## Synthesis and crystallization   

We have succeeded in growing single-crystalline samples of Pr_5_Si_4_ for the first time. For Nd_5_Si_4_, Roger *et al.* (2006 ▸) obtained a very small single crystal, but we have succeeded in growing a large single crystal. These compounds are incongruently melting compounds (Shukla *et al.*, 2009 ▸), so we synthesized source materials with the non-stoichiometric molar ratio of Pr (Nd):Si of 58:42 in a mono-arc furnace. Each melted button of source materials was turned over and remelted three times to ensure homogeneity. Single crystals of Pr_5_Si_4_ and Nd_5_Si_4_ were grown by the Czochralski pulling method in a tetra arc furnace in an argon atmosphere on a water-cooled copper hearth. A tungsten rod was used as a pulling axis with no seed crystal, and after optimizing the initial conditions of the growth, the crystal was pulled at a constant rate of 12 mm hour^−1^. The sizes of the grown ingots were about 30 mm in length and 5 mm in diameter. The grown single-crystal samples were characterized by powder X-ray diffraction using a Rigaku MiniFlexII diffractometer with Cu *K*α radiation. The powder X-ray diffraction peaks can be well indexed based on the tetra­gonal Zr_5_Si_4_-type structure. In addition, it has been confirmed that the whole grown crystal is a single grain crystal by means of the back-reflection Laue method.

## Database survey   

A survey of the Inorganic Crystal Structure Database (ICSD; Belsky *et al.*, 2002 ▸) for Pr_5_Si_4_ yielded three hits. In all three, it is reported that Pr_5_Si_4_ has a Zr_5_Si_4_-type structure (Smith *et al.*, 1967 ▸; ICSD 649362; Yang *et al.*, 2002*b* ▸; ICSD 95099; Yang *et al.*, 2003 ▸; ICSD 98352). On the other hand, for Nd_5_Si_4_, previous reports have shown that Nd_5_Si_4_ has two types of crystal structure, a Sm_5_Ge_4_ type (Raman, 1968 ▸; ICSD 645983; Roger *et al.*, 2006 ▸; ICSD 154658 and 154659) and a Zr_5_Si_4_-type structure (Smith *et al.*, 1967 ▸; ICSD 645939; Mokra *et al.*, 1978 ▸; ICSD 645946; Eremenko *et al.*, 1984 ▸; ICSD 600990; Yang *et al.*, 2002*a* ▸; ICSD 94987; Yang *et al.*, 2002*c* ▸; ICSD 190404; Cadogan *et al.*, 2002 ▸; ICSD 190404). Roger *et al.* (2006 ▸) reported that Sm_5_Ge_4_-type Nd_5_Si_4_ could be obtained only with the addition of a tiny amount of boron of less than three at.% in the initial mixture, and that when synthesized with Nd and Si alone, Zr_5_Si_4_-type Nd_5_Si_4_ was obtained.

## Refinement   

Crystal data, data collection, and structure refinement details are summarized in Table 3 ▸. The highest and deepest remaining difference electron density features are located at 0.90 Å from Pr2 and 1.08 Å from Pr3 for Pr_5_Si_4_, and 0.74 Å from Nd1 and 1.38 Å from Nd2 for Nd_5_Si_4_. The absolute structures of the samples were well-defined in space group *P*4_1_2_1_2 (No. 92), although the bulk samples possibly also contain the other enanti­omer; space group *P*4_3_2_1_2 (No. 96).

## Supplementary Material

Crystal structure: contains datablock(s) global, A, B. DOI: 10.1107/S2056989020002789/ru2068sup1.cif

Structure factors: contains datablock(s) A. DOI: 10.1107/S2056989020002789/ru2068Asup2.hkl

Structure factors: contains datablock(s) B. DOI: 10.1107/S2056989020002789/ru2068Bsup3.hkl

CCDC references: 1974014, 1974013

Additional supporting information:  crystallographic information; 3D view; checkCIF report

## Figures and Tables

**Table 1:** Selected bond lengths (Å) for Pr_5_Si_4_

Pr1—Pr2^i^	3.4914 (4)	Pr1—Si1^ix^	3.1756 (13)
Pr1—Pr2^ii^	3.5319 (4)	Pr1—Si2^ii^	3.1780 (13)
Pr1—Pr2^iii^	3.5319 (4)	Pr1—Si2^iii^	3.1780 (13)
Pr1—Pr2^iv^	3.4914 (4)	Pr2—Pr2^i^	3.9561 (6)
Pr1—Pr3^v^	3.6423 (3)	Pr2—Pr3^vii^	3.9414 (4)
Pr1—Pr3^vi^	3.6423 (3)	Pr2—Pr3^*x*^	3.9717 (3)
Pr1—Si1^vii^	3.1756 (13)	Pr2—Pr3^xi^	3.9156 (3)
Pr1—Si1^viii^	3.0985 (13)	Pr3—Pr3^ii^	4.0074 (2)
Pr1—Si1	3.0985 (13)	Si1—Si2	2.4738 (16)

Symmetry codes: (i) −*y* + 1, −*x* + 1, −*z* + 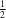; (ii) −*y* + 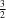, *x* + 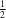, *z* + 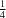; (iii) *x* + 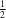, −*y* + 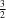, −*z* + 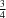; (iv) −*x* + 1, −*y* + 1, *z* + 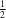; (v) −*y* + 2, −*x* + 1, −*z* + 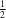; (vi) −*x* + 1, −*y* + 2, *z* + 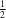; (vii) −*y* + 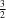, *x* − 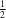, *z* + 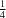; (viii) *y*, *x*, −*z* + 1; (ix) *x* − 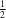, −*y* + 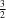, −*z* + 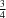.

**Table 2:** Selected bond lengths (Å) for Nd_5_Si_4_

Nd1—Nd2^i^	3.4725 (5)	Nd1—Si1^ix^	3.1528 (16)
Nd1—Nd2^ii^	3.5021 (5)	Nd1—Si2^ii^	3.1661 (16)
Nd1—Nd2^iii^	3.5021 (5)	Nd1—Si2^iii^	3.1661 (16)
Nd1—Nd2^iv^	3.4725 (5)	Nd2—Nd2^i^	3.9202 (7)
Nd1—Nd3^v^	3.6265 (3)	Nd2—Nd3^*x*^	3.9094 (4)
Nd1—Nd3^vi^	3.6265 (3)	Nd2—Nd3^xi^	3.9378 (4)
Nd1—Si1^vii^	3.1528 (16)	Nd2—Nd3^vii^	3.9061 (4)
Nd1—Si1^viii^	3.0744 (15)	Nd3—Nd3^xii^	3.9752 (2)
Nd1—Si1	3.0744 (15)	Si1—Si2	2.482 (2)

Symmetry codes: (i) −*y* + 1, −*x* + 1, −*z* + 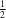; (ii) −*y* + 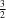, *x* + 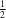, *z* + 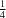; (iii) *x* + 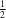, −*y* + 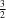, −*z* + 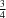; (iv) −*x* + 1, −*y* + 1, *z* + 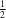; (v) −*y* + 2, −*x* + 1, −*z* + 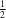; (vi) −*x* + 1, −*y* + 2, *z* + 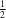; (vii) −*y* + 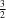, *x* − 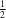, *z* + 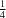; (viii) *y*, *x*, −*z* + 1; (ix) *x* − 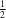, −*y* + 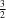, −*z* + 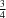.

**Table 3:** Experimental details

	Pr_5_Si_4_	Nd_5_Si_4_
Crystal data		
*M*_r_	816.91	833.56
Crystal system, space group	Tetragonal, *P*4_1_2_1_2	Tetragonal, *P*4_1_2_1_2
Temperature (K)	223	223
*a*, *c* (Å)	7.9001 (2), 14.9568 (6)	7.8644 (2), 14.8085 (5)
*V* (Å^3^)	933.48 (6)	915.89 (6)
*Z*	4	4
Radiation type	Mo *K*α	Mo *K*α
μ (mm^−1^)	26.03	28.27
Crystal size (mm)	0.13 × 0.08 × 0.03	0.12 × 0.09 × 0.07

Data collection		
Diffractometer	XtaLAB AFC12 (RINC): Kappa dual offset/far	XtaLAB AFC12 (RINC): Kappa dual offset/far
Absorption correction	Analytical [*CrysAlis PRO* (Rigaku OD, 2019 ▸) based on Clark & Reid (1995 ▸)]	Analytical [*CrysAlis PRO* (Rigaku OD, 2019 ▸) based on Clark & Reid (1995 ▸)]
*T*_min_, *T*_max_	0.588, 0.830	0.561, 0.702
No. of measured, independent and observed [*I* > 2σ(*I*)] reflections	5553, 1260, 1225	6054, 1238, 1203
*R*_int_	0.025	0.032
(sin θ/λ)_max_ (Å^−1^)	0.710	0.708

Refinement		
*R*[*F* ^2^ > 2σ(*F* ^2^)], *wR*(*F* ^2^), *S*	0.013, 0.026, 1.10	0.015, 0.028, 1.09
No. of reflections	1260	1238
No. of parameters	43	43
Δρ_max_, Δρ_min_ (e Å^−3^)	0.69, −0.85	1.10, −0.71
Absolute structure	Flack *x* determined using 431 quotients [(*I* ^+^)−(*I* ^−^)]/[(*I* ^+^)+(*I* ^−^)] (Parsons *et al.*, 2013 ▸)	Flack *x* determined using 422 quotients [(*I* ^+^)−(*I* ^−^)]/[(*I* ^+^)+(*I* ^−^)] (Parsons *et al.*, 2013 ▸)
Absolute structure parameter	−0.04 (2)	−0.01 (3)

Computer programs: *CrysAlis PRO* (Rigaku OD, 2019 ▸), *SHELXT* (Sheldrick, 2015*a* ▸), *SHELXL* (Sheldrick, 2015*b* ▸), *OLEX2* (Dolomanov *et al.*, 2009 ▸) and *publCIF* (Westrip, 2010 ▸).

## supplementary crystallographic information

### Pentapraseodymium tetrasiliside (A) Crystal data

**Table:**

Pr_5_Si_4_	*D*_x_ = 5.813 Mg m^−^^3^
*M**_r_* = 816.91	Mo *K*α radiation, λ = 0.71073 Å
Tetragonal, *P*4_1_2_1_2	Cell parameters from 4009 reflections
Hall symbol: P 4abw 2nw	θ = 3.8–30.3°
*a* = 7.9001 (2) Å	µ = 26.03 mm^−^^1^
*c* = 14.9568 (6) Å	*T* = 223 K
*V* = 933.48 (6) Å^3^	Plate, metallic gray
*Z* = 4	0.13 × 0.08 × 0.03 mm
*F*(000) = 1404	

### Pentapraseodymium tetrasiliside (A) Data collection

**Table:**

XtaLAB AFC12 (RINC): Kappa dual offset/far diffractometer	1260 independent reflections
Radiation source: micro-focus sealed X-ray tube	1225 reflections with *I* > 2σ(*I*)
Mirror monochromator	*R*_int_ = 0.025
Detector resolution: 5.8140 pixels mm^-1^	θ_max_ = 30.3°, θ_min_ = 3.7°
ω scans	*h* = −11→11
Absorption correction: analytical [CrysAlisPro (Rigaku OD, 2019) based on Clark & Reid (1995)]	*k* = −10→10
*T*_min_ = 0.588, *T*_max_ = 0.830	*l* = −19→17
5553 measured reflections	

### Pentapraseodymium tetrasiliside (A) Refinement

**Table:**

Refinement on *F*^2^	*w* = 1/[σ^2^(*F*_o_^2^) + (0.0078*P*)^2^ + 0.0556*P*] where *P* = (*F*_o_^2^ + 2*F*_c_^2^)/3
Least-squares matrix: full	(Δ/σ)_max_ = 0.001
*R*[*F*^2^ > 2σ(*F*^2^)] = 0.013	Δρ_max_ = 0.69 e Å^−^^3^
*wR*(*F*^2^) = 0.026	Δρ_min_ = −0.85 e Å^−^^3^
*S* = 1.10	Extinction correction: SHELXL (Sheldrick, 2015b), Fc^*^=kFc[1+0.001xFc^2^λ^3^/sin(2θ)]^-1/4^
1260 reflections	Extinction coefficient: 0.00247 (9)
43 parameters	Absolute structure: Flack *x* determined using 431 quotients [(*I*^+^)-(*I*^-^)]/[(*I*^+^)+(*I*^-^)] (Parsons *et al.*, 2013)
0 restraints	Absolute structure parameter: −0.04 (2)
Primary atom site location: dual	

### Pentapraseodymium tetrasiliside (A) Special details

**Table:**

Geometry. All esds (except the esd in the dihedral angle between two l.s. planes) are estimated using the full covariance matrix. The cell esds are taken into account individually in the estimation of esds in distances, angles and torsion angles; correlations between esds in cell parameters are only used when they are defined by crystal symmetry. An approximate (isotropic) treatment of cell esds is used for estimating esds involving l.s. planes.

### Pentapraseodymium tetrasiliside (A) Fractional atomic coordinates and isotropic or equivalent isotropic displacement parameters (Å^2^)

**Table:**

	*x*	*y*	*z*	*U*_iso_*/*U*_eq_	
Pr1	0.81086 (3)	0.81086 (3)	0.500000	0.00721 (8)	
Pr2	0.51578 (3)	0.37028 (3)	0.12478 (2)	0.00578 (7)	
Pr3	0.51088 (3)	0.87144 (3)	0.04758 (2)	0.00676 (7)	
Si1	0.92537 (16)	0.71000 (16)	0.30916 (8)	0.0069 (2)	
Si2	0.69967 (16)	0.66478 (16)	0.19702 (8)	0.0078 (2)	

### Pentapraseodymium tetrasiliside (A) Atomic displacement parameters (Å^2^)

**Table:**

	*U*^11^	*U*^22^	*U*^33^	*U*^12^	*U*^13^	*U*^23^
Pr1	0.00720 (10)	0.00720 (10)	0.00723 (16)	−0.00017 (11)	0.00069 (9)	−0.00069 (9)
Pr2	0.00587 (11)	0.00618 (12)	0.00529 (12)	−0.00033 (9)	0.00050 (9)	−0.00083 (8)
Pr3	0.00630 (12)	0.00650 (12)	0.00748 (12)	−0.00045 (7)	−0.00114 (9)	0.00172 (9)
Si1	0.0074 (5)	0.0078 (6)	0.0056 (6)	−0.0011 (4)	−0.0004 (4)	0.0001 (5)
Si2	0.0066 (5)	0.0074 (6)	0.0094 (6)	−0.0018 (4)	−0.0012 (5)	0.0007 (5)

### Pentapraseodymium tetrasiliside (A) Geometric parameters (Å, º)

**Table:**

Pr1—Pr2^i^	3.4914 (4)	Pr2—Pr3^xi^	3.9156 (3)
Pr1—Pr2^ii^	3.5319 (4)	Pr2—Si1^xi^	3.0641 (13)
Pr1—Pr2^iii^	3.5319 (4)	Pr2—Si1^i^	3.1005 (14)
Pr1—Pr2^iv^	3.4914 (4)	Pr2—Si1^xii^	3.0668 (14)
Pr1—Pr3^v^	3.6423 (3)	Pr2—Si2	2.9480 (13)
Pr1—Pr3^vi^	3.6423 (3)	Pr2—Si2^xi^	2.9737 (13)
Pr1—Si1^vii^	3.1756 (13)	Pr2—Si2^i^	3.0730 (13)
Pr1—Si1^viii^	3.0985 (13)	Pr3—Pr3^ii^	4.0074 (2)
Pr1—Si1	3.0985 (13)	Pr3—Si1^xiii^	3.1554 (13)
Pr1—Si1^ix^	3.1756 (13)	Pr3—Si1^xii^	3.3434 (12)
Pr1—Si2^ii^	3.1780 (13)	Pr3—Si1^xiv^	3.1957 (12)
Pr1—Si2^iii^	3.1780 (13)	Pr3—Si2^xiii^	3.2566 (12)
Pr2—Pr2^i^	3.9561 (6)	Pr3—Si2^xii^	3.1708 (12)
Pr2—Pr3^vii^	3.9414 (4)	Pr3—Si2	3.1442 (12)
Pr2—Pr3^x^	3.9717 (3)	Si1—Si2	2.4738 (16)
			
Pr2^i^—Pr1—Pr2^iv^	71.331 (11)	Si2^xi^—Pr2—Si2^i^	124.47 (4)
Pr2^iii^—Pr1—Pr2^ii^	68.120 (11)	Pr1^xvi^—Pr3—Pr1^xii^	97.461 (6)
Pr2^iv^—Pr1—Pr2^ii^	176.841 (6)	Pr1^xvi^—Pr3—Pr2^x^	99.525 (8)
Pr2^i^—Pr1—Pr2^iii^	176.841 (6)	Pr1^xii^—Pr3—Pr2^xvii^	158.527 (9)
Pr2^i^—Pr1—Pr2^ii^	110.343 (4)	Pr1^xvi^—Pr3—Pr2^xiii^	136.554 (9)
Pr2^iv^—Pr1—Pr2^iii^	110.343 (4)	Pr1^xii^—Pr3—Pr2^x^	97.821 (7)
Pr2^iv^—Pr1—Pr3^v^	109.131 (9)	Pr1^xii^—Pr3—Pr2^xiii^	99.441 (7)
Pr2^iii^—Pr1—Pr3^vi^	74.025 (6)	Pr1^xvi^—Pr3—Pr2^xvii^	102.639 (8)
Pr2^i^—Pr1—Pr3^v^	70.258 (6)	Pr1^xvi^—Pr3—Pr3^ii^	98.905 (6)
Pr2^ii^—Pr1—Pr3^v^	74.025 (6)	Pr1^xii^—Pr3—Pr3^ii^	56.190 (4)
Pr2^ii^—Pr1—Pr3^vi^	106.588 (8)	Pr2^xiii^—Pr3—Pr2^xvii^	60.464 (9)
Pr2^i^—Pr1—Pr3^vi^	109.131 (9)	Pr2^xvii^—Pr3—Pr2^x^	86.316 (6)
Pr2^iv^—Pr1—Pr3^vi^	70.258 (6)	Pr2^xiii^—Pr3—Pr2^x^	117.252 (7)
Pr2^iii^—Pr1—Pr3^v^	106.588 (8)	Pr2^x^—Pr3—Pr3^ii^	149.882 (8)
Pr3^vi^—Pr1—Pr3^v^	179.290 (14)	Pr2^xvii^—Pr3—Pr3^ii^	112.494 (8)
Si1—Pr1—Pr2^iii^	122.55 (3)	Pr2^xiii^—Pr3—Pr3^ii^	60.158 (7)
Si1^viii^—Pr1—Pr2^i^	127.07 (3)	Si1^xii^—Pr3—Pr1^xvi^	89.84 (2)
Si1—Pr1—Pr2^ii^	54.63 (3)	Si1^xiv^—Pr3—Pr1^xii^	54.18 (2)
Si1^ix^—Pr1—Pr2^iii^	128.63 (2)	Si1^xiii^—Pr3—Pr1^xvi^	55.14 (2)
Si1^ix^—Pr1—Pr2^iv^	54.47 (2)	Si1^xii^—Pr3—Pr1^xii^	51.84 (2)
Si1—Pr1—Pr2^i^	55.75 (3)	Si1^xiii^—Pr3—Pr1^xii^	144.17 (3)
Si1^vii^—Pr1—Pr2^iv^	54.52 (2)	Si1^xiv^—Pr3—Pr1^xvi^	53.40 (2)
Si1^viii^—Pr1—Pr2^iii^	54.63 (3)	Si1^xiii^—Pr3—Pr2^xiii^	89.98 (2)
Si1^ix^—Pr1—Pr2^i^	54.52 (2)	Si1^xiv^—Pr3—Pr2^xiii^	108.89 (3)
Si1^vii^—Pr1—Pr2^iii^	124.06 (2)	Si1^xii^—Pr3—Pr2^xvii^	134.80 (2)
Si1^vii^—Pr1—Pr2^i^	54.47 (2)	Si1^xiv^—Pr3—Pr2^x^	129.63 (3)
Si1—Pr1—Pr2^iv^	127.07 (3)	Si1^xii^—Pr3—Pr2^xiii^	131.45 (2)
Si1^viii^—Pr1—Pr2^ii^	122.55 (3)	Si1^xiii^—Pr3—Pr2^xvii^	50.33 (3)
Si1^viii^—Pr1—Pr2^iv^	55.75 (3)	Si1^xii^—Pr3—Pr2^x^	48.60 (2)
Si1^ix^—Pr1—Pr2^ii^	124.06 (2)	Si1^xiii^—Pr3—Pr2^x^	108.41 (2)
Si1^vii^—Pr1—Pr2^ii^	128.63 (2)	Si1^xiv^—Pr3—Pr2^xvii^	136.01 (3)
Si1—Pr1—Pr3^vi^	124.12 (2)	Si1^xiii^—Pr3—Pr3^ii^	101.66 (2)
Si1^viii^—Pr1—Pr3^v^	124.12 (2)	Si1^xii^—Pr3—Pr3^ii^	108.02 (2)
Si1^vii^—Pr1—Pr3^v^	54.62 (2)	Si1^xiv^—Pr3—Pr3^ii^	50.43 (2)
Si1^viii^—Pr1—Pr3^vi^	55.90 (2)	Si1^xiv^—Pr3—Si1^xii^	85.949 (17)
Si1^ix^—Pr1—Pr3^v^	124.76 (3)	Si1^xiii^—Pr3—Si1^xii^	137.237 (18)
Si1^ix^—Pr1—Pr3^vi^	54.62 (2)	Si1^xiii^—Pr3—Si1^xiv^	90.036 (12)
Si1^vii^—Pr1—Pr3^vi^	124.76 (3)	Si1^xiv^—Pr3—Si2^xiii^	90.54 (3)
Si1—Pr1—Pr3^v^	55.90 (2)	Si1^xiii^—Pr3—Si2^xiii^	45.35 (3)
Si1—Pr1—Si1^vii^	91.45 (4)	Si1^xiii^—Pr3—Si2^xii^	92.74 (3)
Si1^viii^—Pr1—Si1^ix^	91.45 (4)	Si2^xii^—Pr3—Pr1^xii^	87.19 (2)
Si1—Pr1—Si1^ix^	90.57 (2)	Si2—Pr3—Pr1^xii^	54.53 (2)
Si1^viii^—Pr1—Si1^vii^	90.57 (2)	Si2—Pr3—Pr1^xvi^	145.87 (2)
Si1—Pr1—Si1^viii^	177.18 (5)	Si2^xiii^—Pr3—Pr1^xii^	123.76 (2)
Si1^ix^—Pr1—Si1^vii^	88.74 (5)	Si2^xiii^—Pr3—Pr1^xvi^	90.26 (2)
Si1^vii^—Pr1—Si2^ii^	178.20 (3)	Si2^xii^—Pr3—Pr1^xvi^	55.08 (2)
Si1^ix^—Pr1—Si2^ii^	92.22 (3)	Si2^xiii^—Pr3—Pr2^xvii^	49.43 (2)
Si1^vii^—Pr1—Si2^iii^	92.22 (3)	Si2^xiii^—Pr3—Pr2^xiii^	47.45 (2)
Si1—Pr1—Si2^ii^	90.07 (3)	Si2^xiii^—Pr3—Pr2^x^	135.71 (2)
Si1^ix^—Pr1—Si2^iii^	178.20 (3)	Si2^xii^—Pr3—Pr2^xiii^	164.53 (2)
Si1—Pr1—Si2^iii^	87.88 (3)	Si2—Pr3—Pr2^xiii^	48.31 (2)
Si1^viii^—Pr1—Si2^iii^	90.07 (3)	Si2—Pr3—Pr2^xvii^	103.99 (2)
Si1^viii^—Pr1—Si2^ii^	87.88 (3)	Si2^xii^—Pr3—Pr2^xvii^	110.50 (2)
Si2^iii^—Pr1—Pr2^ii^	54.20 (2)	Si2—Pr3—Pr2^x^	103.11 (2)
Si2^iii^—Pr1—Pr2^iv^	127.29 (2)	Si2^xii^—Pr3—Pr2^x^	47.59 (2)
Si2^ii^—Pr1—Pr2^ii^	51.80 (2)	Si2^xiii^—Pr3—Pr3^ii^	67.57 (2)
Si2^iii^—Pr1—Pr2^i^	125.04 (2)	Si2—Pr3—Pr3^ii^	50.90 (2)
Si2^ii^—Pr1—Pr2^iv^	125.04 (2)	Si2^xii^—Pr3—Pr3^ii^	133.74 (2)
Si2^ii^—Pr1—Pr2^iii^	54.20 (2)	Si2^xii^—Pr3—Si1^xiv^	86.34 (3)
Si2^iii^—Pr1—Pr2^iii^	51.80 (2)	Si2—Pr3—Si1^xii^	86.35 (3)
Si2^ii^—Pr1—Pr2^i^	127.29 (2)	Si2^xiii^—Pr3—Si1^xii^	175.55 (3)
Si2^iii^—Pr1—Pr3^vi^	125.73 (2)	Si2^xii^—Pr3—Si1^xii^	44.54 (3)
Si2^ii^—Pr1—Pr3^vi^	54.90 (2)	Si2—Pr3—Si1^xiv^	92.48 (3)
Si2^ii^—Pr1—Pr3^v^	125.73 (2)	Si2—Pr3—Si1^xiii^	136.40 (3)
Si2^iii^—Pr1—Pr3^v^	54.90 (2)	Si2—Pr3—Si2^xiii^	91.079 (17)
Si2^iii^—Pr1—Si2^ii^	86.85 (5)	Si2^xii^—Pr3—Si2^xiii^	138.014 (18)
Pr1^xv^—Pr2—Pr1^xii^	103.710 (6)	Si2—Pr3—Si2^xii^	130.86 (2)
Pr1^xii^—Pr2—Pr2^i^	55.940 (5)	Pr1—Si1—Pr1^xvii^	123.37 (4)
Pr1^xv^—Pr2—Pr2^i^	140.135 (10)	Pr1—Si1—Pr2^i^	68.56 (3)
Pr1^xv^—Pr2—Pr3^vii^	102.847 (8)	Pr1—Si1—Pr3^ii^	70.13 (2)
Pr1^xv^—Pr2—Pr3^x^	106.888 (8)	Pr1^xvii^—Si1—Pr3^ii^	134.03 (4)
Pr1^xii^—Pr2—Pr3^xi^	106.191 (8)	Pr1^xvii^—Si1—Pr3^v^	71.14 (3)
Pr1^xii^—Pr2—Pr3^vii^	105.646 (8)	Pr1—Si1—Pr3^v^	70.70 (3)
Pr1^xii^—Pr2—Pr3^x^	117.969 (7)	Pr1—Si1—Pr3^xi^	136.94 (4)
Pr1^xv^—Pr2—Pr3^xi^	149.920 (8)	Pr2^xiii^—Si1—Pr1^xvii^	68.02 (3)
Pr2^i^—Pr2—Pr3^x^	112.953 (9)	Pr2^ii^—Si1—Pr1	69.90 (3)
Pr3^vii^—Pr2—Pr2^i^	59.445 (6)	Pr2^xiii^—Si1—Pr1	139.92 (4)
Pr3^xi^—Pr2—Pr2^i^	60.092 (6)	Pr2^ii^—Si1—Pr1^xvii^	67.99 (3)
Pr3^xi^—Pr2—Pr3^x^	61.068 (5)	Pr2^i^—Si1—Pr1^xvii^	141.38 (4)
Pr3^xi^—Pr2—Pr3^vii^	65.880 (8)	Pr2^xiii^—Si1—Pr2^i^	129.66 (4)
Pr3^vii^—Pr2—Pr3^x^	117.851 (7)	Pr2^ii^—Si1—Pr2^i^	138.39 (4)
Si1^xi^—Pr2—Pr1^xv^	57.51 (2)	Pr2^xiii^—Si1—Pr2^ii^	83.22 (3)
Si1^i^—Pr2—Pr1^xii^	96.00 (2)	Pr2^xiii^—Si1—Pr3^xi^	82.63 (3)
Si1^xii^—Pr2—Pr1^xii^	55.47 (2)	Pr2^xiii^—Si1—Pr3^ii^	76.47 (3)
Si1^xii^—Pr2—Pr1^xv^	57.49 (2)	Pr2^ii^—Si1—Pr3^xi^	138.21 (4)
Si1^i^—Pr2—Pr1^xv^	55.69 (2)	Pr2^ii^—Si1—Pr3^v^	87.20 (3)
Si1^xi^—Pr2—Pr1^xii^	147.18 (3)	Pr2^ii^—Si1—Pr3^ii^	79.97 (3)
Si1^xii^—Pr2—Pr2^i^	111.25 (2)	Pr2^i^—Si1—Pr3^xi^	78.10 (3)
Si1^xi^—Pr2—Pr2^i^	155.46 (3)	Pr2^i^—Si1—Pr3^ii^	84.10 (3)
Si1^i^—Pr2—Pr2^i^	90.03 (3)	Pr2^i^—Si1—Pr3^v^	81.41 (3)
Si1^xi^—Pr2—Pr3^vii^	104.88 (3)	Pr2^xiii^—Si1—Pr3^v^	138.72 (4)
Si1^xi^—Pr2—Pr3^x^	54.93 (2)	Pr3^xi^—Si1—Pr1^xvii^	70.24 (3)
Si1^i^—Pr2—Pr3^x^	145.54 (2)	Pr3^xi^—Si1—Pr3^v^	78.24 (3)
Si1^i^—Pr2—Pr3^vii^	51.57 (2)	Pr3^xi^—Si1—Pr3^ii^	133.50 (4)
Si1^xi^—Pr2—Pr3^xi^	97.03 (3)	Pr3^v^—Si1—Pr3^ii^	140.82 (4)
Si1^xii^—Pr2—Pr3^x^	101.67 (2)	Si2—Si1—Pr1	116.82 (5)
Si1^xii^—Pr2—Pr3^xi^	147.77 (2)	Si2—Si1—Pr1^xvii^	119.66 (5)
Si1^i^—Pr2—Pr3^xi^	117.17 (2)	Si2—Si1—Pr2^xiii^	63.92 (4)
Si1^xii^—Pr2—Pr3^vii^	140.10 (2)	Si2—Si1—Pr2^ii^	135.25 (5)
Si1^xii^—Pr2—Si1^i^	92.601 (18)	Si2—Si1—Pr2^i^	65.79 (4)
Si1^xi^—Pr2—Si1^i^	93.56 (3)	Si2—Si1—Pr3^v^	137.54 (6)
Si1^xi^—Pr2—Si1^xii^	92.85 (4)	Si2—Si1—Pr3^ii^	64.03 (4)
Si1^xi^—Pr2—Si2^i^	140.79 (3)	Si2—Si1—Pr3^xi^	69.49 (4)
Si1^xii^—Pr2—Si2^i^	90.38 (3)	Pr1^xii^—Si2—Pr3^xi^	135.69 (4)
Si2^i^—Pr2—Pr1^xv^	92.68 (2)	Pr2—Si2—Pr1^xii^	70.30 (3)
Si2—Pr2—Pr1^xii^	57.90 (2)	Pr2^xiii^—Si2—Pr1^xii^	141.97 (4)
Si2^xi^—Pr2—Pr1^xii^	158.04 (2)	Pr2^i^—Si2—Pr1^xii^	68.78 (3)
Si2^i^—Pr2—Pr1^xii^	57.02 (2)	Pr2—Si2—Pr2^xiii^	132.15 (4)
Si2—Pr2—Pr1^xv^	152.08 (2)	Pr2—Si2—Pr2^i^	82.12 (3)
Si2^xi^—Pr2—Pr1^xv^	98.16 (2)	Pr2^xiii^—Si2—Pr2^i^	134.64 (4)
Si2^xi^—Pr2—Pr2^i^	107.11 (2)	Pr2^xiii^—Si2—Pr3^ii^	80.47 (3)
Si2—Pr2—Pr2^i^	50.30 (2)	Pr2^xiii^—Si2—Pr3^xi^	82.34 (3)
Si2^i^—Pr2—Pr2^i^	47.57 (2)	Pr2—Si2—Pr3^ii^	140.09 (4)
Si2^i^—Pr2—Pr3^vii^	53.61 (2)	Pr2^i^—Si2—Pr3^ii^	87.54 (3)
Si2^xi^—Pr2—Pr3^x^	51.94 (2)	Pr2—Si2—Pr3^xi^	78.08 (3)
Si2^xi^—Pr2—Pr3^xi^	52.15 (2)	Pr2^i^—Si2—Pr3^xi^	76.97 (3)
Si2^xi^—Pr2—Pr3^vii^	70.89 (2)	Pr2^xiii^—Si2—Pr3	79.53 (3)
Si2^i^—Pr2—Pr3^xi^	100.75 (2)	Pr2—Si2—Pr3	85.15 (3)
Si2—Pr2—Pr3^xi^	54.47 (2)	Pr2^i^—Si2—Pr3	140.56 (4)
Si2^i^—Pr2—Pr3^x^	160.33 (3)	Pr3—Si2—Pr1^xii^	71.78 (3)
Si2—Pr2—Pr3^x^	70.64 (3)	Pr3^ii^—Si2—Pr1^xii^	70.02 (3)
Si2—Pr2—Pr3^vii^	102.52 (2)	Pr3—Si2—Pr3^xi^	136.07 (4)
Si2—Pr2—Si1^xii^	95.16 (3)	Pr3—Si2—Pr3^ii^	78.78 (3)
Si2—Pr2—Si1^i^	139.57 (4)	Pr3^ii^—Si2—Pr3^xi^	136.58 (4)
Si2^i^—Pr2—Si1^i^	47.24 (3)	Si1—Si2—Pr1^xii^	121.26 (5)
Si2^xi^—Pr2—Si1^xii^	140.11 (3)	Si1—Si2—Pr2^i^	66.96 (4)
Si2^xi^—Pr2—Si1^i^	97.97 (3)	Si1—Si2—Pr2^xiii^	67.74 (4)
Si2^xi^—Pr2—Si1^xi^	48.35 (3)	Si1—Si2—Pr2	135.86 (5)
Si2—Pr2—Si1^xi^	125.48 (3)	Si1—Si2—Pr3^ii^	71.43 (4)
Si2—Pr2—Si2^xi^	100.995 (18)	Si1—Si2—Pr3	138.49 (5)
Si2—Pr2—Si2^i^	93.03 (4)	Si1—Si2—Pr3^xi^	65.16 (4)

Symmetry codes: (i) −*y*+1, −*x*+1, −*z*+1/2; (ii) −*y*+3/2, *x*+1/2, *z*+1/4; (iii) *x*+1/2, −*y*+3/2, −*z*+3/4; (iv) −*x*+1, −*y*+1, *z*+1/2; (v) −*y*+2, −*x*+1, −*z*+1/2; (vi) −*x*+1, −*y*+2, *z*+1/2; (vii) −*y*+3/2, *x*−1/2, *z*+1/4; (viii) *y*, *x*, −*z*+1; (ix) *x*−1/2, −*y*+3/2, −*z*+3/4; (x) *y*, *x*, −*z*; (xi) −*x*+3/2, *y*−1/2, −*z*+1/4; (xii) *y*−1/2, −*x*+3/2, *z*−1/4; (xiii) −*x*+3/2, *y*+1/2, −*z*+1/4; (xiv) −*y*+1, −*x*+2, −*z*+1/2; (xv) −*x*+1, −*y*+1, *z*−1/2; (xvi) −*x*+1, −*y*+2, *z*−1/2; (xvii) *y*+1/2, −*x*+3/2, *z*−1/4.

### Pentaneodymium tetrasiliside (B) Crystal data

**Table:**

Nd_5_Si_4_	*D*_x_ = 6.045 Mg m^−^^3^
*M**_r_* = 833.56	Mo *K*α radiation, λ = 0.71073 Å
Tetragonal, *P*4_1_2_1_2	Cell parameters from 3985 reflections
Hall symbol: P 4abw 2nw	θ = 3.8–30.4°
*a* = 7.8644 (2) Å	µ = 28.27 mm^−^^1^
*c* = 14.8085 (5) Å	*T* = 223 K
*V* = 915.89 (6) Å^3^	Plate, metallic gray
*Z* = 4	0.12 × 0.09 × 0.07 mm
*F*(000) = 1424	

### Pentaneodymium tetrasiliside (B) Data collection

**Table:**

XtaLAB AFC12 (RINC): Kappa dual offset/far diffractometer	1238 independent reflections
Radiation source: micro-focus sealed X-ray tube	1203 reflections with *I* > 2σ(*I*)
Mirror monochromator	*R*_int_ = 0.032
Detector resolution: 5.8140 pixels mm^-1^	θ_max_ = 30.2°, θ_min_ = 3.7°
ω scans	*h* = −11→10
Absorption correction: analytical [CrysAlisPro (Rigaku OD, 2019) based on Clark & Reid (1995)]	*k* = −10→10
*T*_min_ = 0.561, *T*_max_ = 0.702	*l* = −20→19
6054 measured reflections	

### Pentaneodymium tetrasiliside (B) Refinement

**Table:**

Refinement on *F*^2^	*w* = 1/[σ^2^(*F*_o_^2^) + 0.4858*P*] where *P* = (*F*_o_^2^ + 2*F*_c_^2^)/3
Least-squares matrix: full	(Δ/σ)_max_ = 0.001
*R*[*F*^2^ > 2σ(*F*^2^)] = 0.015	Δρ_max_ = 1.10 e Å^−^^3^
*wR*(*F*^2^) = 0.028	Δρ_min_ = −0.71 e Å^−^^3^
*S* = 1.09	Extinction correction: SHELXL (Sheldrick, 2015b), Fc^*^=kFc[1+0.001xFc^2^λ^3^/sin(2θ)]^-1/4^
1238 reflections	Extinction coefficient: 0.00090 (6)
43 parameters	Absolute structure: Flack *x* determined using 422 quotients [(*I*^+^)-(*I*^-^)]/[(*I*^+^)+(*I*^-^)] (Parsons *et al.*, 2013)
0 restraints	Absolute structure parameter: −0.01 (3)
Primary atom site location: dual	

### Pentaneodymium tetrasiliside (B) Special details

**Table:**

Geometry. All esds (except the esd in the dihedral angle between two l.s. planes) are estimated using the full covariance matrix. The cell esds are taken into account individually in the estimation of esds in distances, angles and torsion angles; correlations between esds in cell parameters are only used when they are defined by crystal symmetry. An approximate (isotropic) treatment of cell esds is used for estimating esds involving l.s. planes.

### Pentaneodymium tetrasiliside (B) Fractional atomic coordinates and isotropic or equivalent isotropic displacement parameters (Å^2^)

**Table:**

	*x*	*y*	*z*	*U*_iso_*/*U*_eq_	
Nd1	0.81184 (4)	0.81184 (4)	0.500000	0.00637 (9)	
Nd2	0.51490 (3)	0.36933 (4)	0.12498 (2)	0.00518 (7)	
Nd3	0.51034 (4)	0.87021 (4)	0.04676 (2)	0.00649 (7)	
Si1	0.9274 (2)	0.7098 (2)	0.30921 (10)	0.0060 (3)	
Si2	0.7003 (2)	0.6636 (2)	0.19543 (10)	0.0078 (3)	

### Pentaneodymium tetrasiliside (B) Atomic displacement parameters (Å^2^)

**Table:**

	*U*^11^	*U*^22^	*U*^33^	*U*^12^	*U*^13^	*U*^23^
Nd1	0.00630 (11)	0.00630 (11)	0.00652 (17)	−0.00026 (13)	0.00064 (11)	−0.00064 (11)
Nd2	0.00538 (14)	0.00534 (14)	0.00483 (12)	0.00002 (12)	0.00035 (11)	−0.00097 (10)
Nd3	0.00617 (15)	0.00631 (14)	0.00700 (13)	−0.00027 (9)	−0.00098 (11)	0.00152 (11)
Si1	0.0060 (7)	0.0066 (7)	0.0054 (6)	−0.0007 (5)	0.0001 (5)	0.0001 (6)
Si2	0.0069 (7)	0.0073 (7)	0.0093 (6)	−0.0016 (5)	−0.0015 (6)	0.0017 (6)

### Pentaneodymium tetrasiliside (B) Geometric parameters (Å, º)

**Table:**

Nd1—Nd2^i^	3.4725 (5)	Nd2—Nd3^vii^	3.9061 (4)
Nd1—Nd2^ii^	3.5021 (5)	Nd2—Si1^x^	3.0366 (16)
Nd1—Nd2^iii^	3.5021 (5)	Nd2—Si1^i^	3.0848 (17)
Nd1—Nd2^iv^	3.4725 (5)	Nd2—Si1^xii^	3.0436 (17)
Nd1—Nd3^v^	3.6265 (3)	Nd2—Si2	2.9272 (17)
Nd1—Nd3^vi^	3.6265 (3)	Nd2—Si2^x^	2.9533 (17)
Nd1—Si1^vii^	3.1528 (16)	Nd2—Si2^i^	3.0565 (15)
Nd1—Si1^viii^	3.0744 (15)	Nd3—Nd3^xii^	3.9752 (2)
Nd1—Si1	3.0744 (15)	Nd3—Si1^xiii^	3.1359 (15)
Nd1—Si1^ix^	3.1528 (16)	Nd3—Si1^xii^	3.3315 (14)
Nd1—Si2^ii^	3.1661 (16)	Nd3—Si1^xiv^	3.1748 (15)
Nd1—Si2^iii^	3.1661 (16)	Nd3—Si2^xiii^	3.2425 (15)
Nd2—Nd2^i^	3.9202 (7)	Nd3—Si2^xii^	3.1619 (16)
Nd2—Nd3^x^	3.9094 (4)	Nd3—Si2	3.1177 (14)
Nd2—Nd3^xi^	3.9378 (4)	Si1—Si2	2.482 (2)
			
Nd2^i^—Nd1—Nd2^iv^	71.144 (14)	Si2^x^—Nd2—Si2^i^	123.86 (5)
Nd2^iii^—Nd1—Nd2^ii^	68.070 (13)	Nd1^xvi^—Nd3—Nd1^xii^	97.355 (7)
Nd2^iv^—Nd1—Nd2^ii^	177.032 (7)	Nd1^xvi^—Nd3—Nd2^xi^	99.740 (9)
Nd2^i^—Nd1—Nd2^iii^	177.032 (7)	Nd1^xii^—Nd3—Nd2^xiii^	99.322 (9)
Nd2^i^—Nd1—Nd2^ii^	110.453 (5)	Nd1^xvi^—Nd3—Nd2^xvii^	102.777 (9)
Nd2^iv^—Nd1—Nd2^iii^	110.453 (5)	Nd1^xii^—Nd3—Nd2^xi^	97.621 (8)
Nd2^iv^—Nd1—Nd3^v^	70.374 (8)	Nd1^xii^—Nd3—Nd2^xvii^	158.298 (11)
Nd2^iii^—Nd1—Nd3^vi^	106.665 (10)	Nd1^xvi^—Nd3—Nd2^xiii^	136.478 (12)
Nd2^i^—Nd1—Nd3^v^	108.985 (11)	Nd1^xvi^—Nd3—Nd3^xii^	57.694 (6)
Nd2^ii^—Nd1—Nd3^v^	106.665 (10)	Nd1^xii^—Nd3—Nd3^xii^	137.385 (10)
Nd2^ii^—Nd1—Nd3^vi^	73.978 (8)	Nd2^xvii^—Nd3—Nd2^xiii^	60.212 (10)
Nd2^i^—Nd1—Nd3^vi^	70.374 (8)	Nd2^xiii^—Nd3—Nd2^xi^	117.333 (8)
Nd2^iv^—Nd1—Nd3^vi^	108.985 (11)	Nd2^xvii^—Nd3—Nd2^xi^	86.968 (8)
Nd2^iii^—Nd1—Nd3^v^	73.978 (8)	Nd2^xi^—Nd3—Nd3^xii^	59.212 (7)
Nd3^vi^—Nd1—Nd3^v^	179.256 (17)	Nd2^xiii^—Nd3—Nd3^xii^	122.770 (9)
Si1—Nd1—Nd2^iii^	122.55 (4)	Nd2^xvii^—Nd3—Nd3^xii^	62.562 (7)
Si1^viii^—Nd1—Nd2^i^	126.97 (4)	Si1^xii^—Nd3—Nd1^xvi^	89.95 (3)
Si1—Nd1—Nd2^ii^	54.67 (3)	Si1^xiv^—Nd3—Nd1^xii^	54.16 (3)
Si1^ix^—Nd1—Nd2^iii^	128.53 (3)	Si1^xiii^—Nd3—Nd1^xvi^	55.00 (3)
Si1^ix^—Nd1—Nd2^iv^	54.30 (3)	Si1^xii^—Nd3—Nd1^xii^	51.72 (3)
Si1—Nd1—Nd2^i^	55.82 (3)	Si1^xiii^—Nd3—Nd1^xii^	143.78 (3)
Si1^vii^—Nd1—Nd2^iv^	54.44 (3)	Si1^xiv^—Nd3—Nd1^xvi^	53.25 (3)
Si1^viii^—Nd1—Nd2^iii^	54.67 (3)	Si1^xiii^—Nd3—Nd2^xvii^	50.52 (3)
Si1^ix^—Nd1—Nd2^i^	54.44 (3)	Si1^xiv^—Nd3—Nd2^xvii^	135.69 (3)
Si1^vii^—Nd1—Nd2^iii^	124.35 (3)	Si1^xii^—Nd3—Nd2^xiii^	131.30 (3)
Si1^vii^—Nd1—Nd2^i^	54.30 (3)	Si1^xiv^—Nd3—Nd2^xi^	129.50 (3)
Si1—Nd1—Nd2^iv^	126.97 (4)	Si1^xii^—Nd3—Nd2^xvii^	135.35 (3)
Si1^viii^—Nd1—Nd2^ii^	122.55 (4)	Si1^xiii^—Nd3—Nd2^xiii^	90.04 (3)
Si1^viii^—Nd1—Nd2^iv^	55.82 (3)	Si1^xii^—Nd3—Nd2^xi^	48.50 (3)
Si1^ix^—Nd1—Nd2^ii^	124.35 (3)	Si1^xiii^—Nd3—Nd2^xi^	108.97 (3)
Si1^vii^—Nd1—Nd2^ii^	128.53 (3)	Si1^xiv^—Nd3—Nd2^xiii^	108.81 (3)
Si1—Nd1—Nd3^vi^	55.83 (3)	Si1^xiii^—Nd3—Nd3^xii^	51.39 (3)
Si1^viii^—Nd1—Nd3^v^	55.83 (3)	Si1^xii^—Nd3—Nd3^xii^	91.19 (3)
Si1^vii^—Nd1—Nd3^v^	124.78 (3)	Si1^xiv^—Nd3—Nd3^xii^	110.85 (3)
Si1^viii^—Nd1—Nd3^vi^	124.19 (3)	Si1^xiv^—Nd3—Si1^xii^	85.88 (2)
Si1^ix^—Nd1—Nd3^v^	54.57 (3)	Si1^xiii^—Nd3—Si1^xii^	137.46 (2)
Si1^ix^—Nd1—Nd3^vi^	124.78 (3)	Si1^xiii^—Nd3—Si1^xiv^	89.678 (15)
Si1^vii^—Nd1—Nd3^vi^	54.57 (3)	Si1^xiv^—Nd3—Si2^xiii^	90.11 (4)
Si1—Nd1—Nd3^v^	124.19 (3)	Si1^xiii^—Nd3—Si2^xiii^	45.76 (4)
Si1—Nd1—Si1^vii^	91.21 (4)	Si1^xiii^—Nd3—Si2^xii^	92.67 (4)
Si1^viii^—Nd1—Si1^ix^	91.21 (4)	Si2^xii^—Nd3—Nd1^xii^	87.36 (3)
Si1—Nd1—Si1^ix^	90.79 (3)	Si2—Nd3—Nd1^xii^	54.76 (3)
Si1^viii^—Nd1—Si1^vii^	90.79 (3)	Si2—Nd3—Nd1^xvi^	146.00 (3)
Si1—Nd1—Si1^viii^	177.21 (7)	Si2^xiii^—Nd3—Nd1^xii^	123.04 (3)
Si1^ix^—Nd1—Si1^vii^	88.51 (6)	Si2^xiii^—Nd3—Nd1^xvi^	90.38 (3)
Si1^vii^—Nd1—Si2^ii^	178.58 (4)	Si2^xii^—Nd3—Nd1^xvi^	55.09 (3)
Si1^ix^—Nd1—Si2^ii^	92.27 (4)	Si2^xiii^—Nd3—Nd2^xiii^	47.19 (3)
Si1^vii^—Nd1—Si2^iii^	92.27 (4)	Si2^xiii^—Nd3—Nd2^xvii^	49.57 (3)
Si1—Nd1—Si2^ii^	89.96 (4)	Si2^xiii^—Nd3—Nd2^xi^	136.52 (3)
Si1^ix^—Nd1—Si2^iii^	178.58 (4)	Si2^xii^—Nd3—Nd2^xvii^	110.79 (3)
Si1—Nd1—Si2^iii^	88.01 (4)	Si2—Nd3—Nd2^xvii^	103.54 (3)
Si1^viii^—Nd1—Si2^iii^	89.96 (4)	Si2—Nd3—Nd2^xiii^	48.09 (3)
Si1^viii^—Nd1—Si2^ii^	88.01 (4)	Si2^xii^—Nd3—Nd2^xiii^	164.61 (3)
Si2^iii^—Nd1—Nd2^ii^	54.28 (3)	Si2—Nd3—Nd2^xi^	102.77 (3)
Si2^iii^—Nd1—Nd2^iv^	127.09 (3)	Si2^xii^—Nd3—Nd2^xi^	47.63 (3)
Si2^ii^—Nd1—Nd2^ii^	51.78 (3)	Si2^xiii^—Nd3—Nd3^xii^	93.42 (3)
Si2^iii^—Nd1—Nd2^i^	125.25 (3)	Si2—Nd3—Nd3^xii^	156.02 (3)
Si2^ii^—Nd1—Nd2^iv^	125.25 (3)	Si2^xii^—Nd3—Nd3^xii^	50.23 (3)
Si2^ii^—Nd1—Nd2^iii^	54.28 (3)	Si2^xii^—Nd3—Si1^xiv^	86.36 (4)
Si2^iii^—Nd1—Nd2^iii^	51.78 (3)	Si2—Nd3—Si1^xii^	86.26 (4)
Si2^ii^—Nd1—Nd2^i^	127.09 (3)	Si2^xiii^—Nd3—Si1^xii^	174.74 (4)
Si2^iii^—Nd1—Nd3^vi^	54.98 (3)	Si2^xii^—Nd3—Si1^xii^	44.85 (4)
Si2^ii^—Nd1—Nd3^vi^	125.68 (3)	Si2—Nd3—Si1^xiv^	92.77 (4)
Si2^ii^—Nd1—Nd3^v^	54.98 (3)	Si2—Nd3—Si1^xiii^	136.26 (4)
Si2^iii^—Nd1—Nd3^v^	125.68 (3)	Si2—Nd3—Si2^xiii^	90.54 (2)
Si2^iii^—Nd1—Si2^ii^	86.97 (6)	Si2^xii^—Nd3—Si2^xiii^	138.34 (2)
Nd1^xv^—Nd2—Nd1^xii^	103.774 (8)	Si2—Nd3—Si2^xii^	131.07 (3)
Nd1^xii^—Nd2—Nd2^i^	55.965 (6)	Nd1—Si1—Nd1^xvii^	123.58 (5)
Nd1^xv^—Nd2—Nd2^i^	140.405 (13)	Nd1—Si1—Nd2^i^	68.64 (3)
Nd1^xv^—Nd2—Nd3^x^	149.935 (9)	Nd1—Si1—Nd3^ii^	70.00 (3)
Nd1^xv^—Nd2—Nd3^xi^	107.244 (10)	Nd1^xvii^—Si1—Nd3^ii^	134.15 (5)
Nd1^xii^—Nd2—Nd3^vii^	106.182 (10)	Nd1^xvii^—Si1—Nd3^vi^	71.13 (3)
Nd1^xii^—Nd2—Nd3^x^	106.111 (9)	Nd1—Si1—Nd3^vi^	70.93 (3)
Nd1^xii^—Nd2—Nd3^xi^	117.604 (9)	Nd1—Si1—Nd3^x^	136.75 (6)
Nd1^xv^—Nd2—Nd3^vii^	102.810 (9)	Nd2^xiii^—Si1—Nd1^xvii^	68.23 (3)
Nd2^i^—Nd2—Nd3^xi^	112.321 (11)	Nd2^ii^—Si1—Nd1	69.84 (3)
Nd3^x^—Nd2—Nd2^i^	59.853 (7)	Nd2^xiii^—Si1—Nd1	139.62 (5)
Nd3^vii^—Nd2—Nd2^i^	59.937 (8)	Nd2^ii^—Si1—Nd1^xvii^	68.14 (4)
Nd3^vii^—Nd2—Nd3^xi^	117.388 (8)	Nd2^i^—Si1—Nd1^xvii^	141.48 (5)
Nd3^vii^—Nd2—Nd3^x^	65.627 (10)	Nd2^xiii^—Si1—Nd2^i^	129.31 (5)
Nd3^x^—Nd2—Nd3^xi^	60.872 (7)	Nd2^ii^—Si1—Nd2^i^	138.41 (5)
Si1^x^—Nd2—Nd1^xv^	57.47 (3)	Nd2^xiii^—Si1—Nd2^ii^	83.28 (4)
Si1^i^—Nd2—Nd1^xii^	96.51 (3)	Nd2^xiii^—Si1—Nd3^x^	83.05 (4)
Si1^xii^—Nd2—Nd1^xii^	55.49 (3)	Nd2^xiii^—Si1—Nd3^ii^	76.24 (3)
Si1^xii^—Nd2—Nd1^xv^	57.42 (3)	Nd2^ii^—Si1—Nd3^x^	138.55 (5)
Si1^i^—Nd2—Nd1^xv^	55.54 (3)	Nd2^ii^—Si1—Nd3^vi^	87.22 (4)
Si1^x^—Nd2—Nd1^xii^	147.02 (3)	Nd2^ii^—Si1—Nd3^ii^	80.06 (4)
Si1^xii^—Nd2—Nd2^i^	111.31 (3)	Nd2^i^—Si1—Nd3^x^	77.79 (4)
Si1^x^—Nd2—Nd2^i^	155.46 (3)	Nd2^i^—Si1—Nd3^ii^	83.83 (4)
Si1^i^—Nd2—Nd2^i^	90.59 (3)	Nd2^i^—Si1—Nd3^vi^	81.65 (4)
Si1^x^—Nd2—Nd3^x^	97.14 (3)	Nd2^xiii^—Si1—Nd3^vi^	138.92 (5)
Si1^x^—Nd2—Nd3^xi^	55.26 (3)	Nd3^x^—Si1—Nd1^xvii^	70.43 (3)
Si1^i^—Nd2—Nd3^xi^	145.44 (3)	Nd3^x^—Si1—Nd3^vi^	78.09 (3)
Si1^i^—Nd2—Nd3^x^	117.05 (3)	Nd3^x^—Si1—Nd3^ii^	133.33 (5)
Si1^x^—Nd2—Nd3^vii^	104.58 (3)	Nd3^vi^—Si1—Nd3^ii^	140.92 (5)
Si1^xii^—Nd2—Nd3^xi^	101.82 (3)	Si2—Si1—Nd1	116.71 (6)
Si1^xii^—Nd2—Nd3^vii^	140.39 (3)	Si2—Si1—Nd1^xvii^	119.56 (6)
Si1^i^—Nd2—Nd3^vii^	51.69 (3)	Si2—Si1—Nd2^xiii^	63.78 (5)
Si1^xii^—Nd2—Nd3^x^	147.88 (3)	Si2—Si1—Nd2^ii^	135.29 (6)
Si1^xii^—Nd2—Si1^i^	92.69 (2)	Si2—Si1—Nd2^i^	65.57 (5)
Si1^x^—Nd2—Si1^i^	93.26 (4)	Si2—Si1—Nd3^vi^	137.48 (7)
Si1^x^—Nd2—Si1^xii^	92.72 (5)	Si2—Si1—Nd3^ii^	63.95 (4)
Si1^x^—Nd2—Si2^i^	140.91 (4)	Si2—Si1—Nd3^x^	69.39 (5)
Si1^xii^—Nd2—Si2^i^	90.60 (4)	Nd1^xii^—Si2—Nd3^x^	135.36 (6)
Si2^i^—Nd2—Nd1^xv^	92.89 (3)	Nd2—Si2—Nd1^xii^	70.04 (4)
Si2—Nd2—Nd1^xii^	58.18 (3)	Nd2^xiii^—Si2—Nd1^xii^	142.04 (5)
Si2^x^—Nd2—Nd1^xii^	157.63 (3)	Nd2^i^—Si2—Nd1^xii^	68.47 (3)
Si2^i^—Nd2—Nd1^xii^	57.24 (3)	Nd2—Si2—Nd2^xiii^	133.24 (5)
Si2—Nd2—Nd1^xv^	151.99 (3)	Nd2—Si2—Nd2^i^	81.83 (4)
Si2^x^—Nd2—Nd1^xv^	98.52 (3)	Nd2^xiii^—Si2—Nd2^i^	134.00 (5)
Si2^x^—Nd2—Nd2^i^	106.53 (3)	Nd2^xiii^—Si2—Nd3^ii^	80.09 (4)
Si2—Nd2—Nd2^i^	50.51 (3)	Nd2^xiii^—Si2—Nd3^x^	82.54 (4)
Si2^i^—Nd2—Nd2^i^	47.66 (3)	Nd2—Si2—Nd3^ii^	139.78 (6)
Si2^i^—Nd2—Nd3^x^	100.42 (3)	Nd2^i^—Si2—Nd3^ii^	87.23 (4)
Si2^x^—Nd2—Nd3^xi^	52.28 (3)	Nd2—Si2—Nd3^x^	78.46 (4)
Si2^x^—Nd2—Nd3^vii^	70.03 (3)	Nd2^i^—Si2—Nd3^x^	76.59 (3)
Si2^x^—Nd2—Nd3^x^	51.78 (3)	Nd2^xiii^—Si2—Nd3	80.12 (4)
Si2^i^—Nd2—Nd3^vii^	53.85 (3)	Nd2—Si2—Nd3	85.51 (4)
Si2—Nd2—Nd3^vii^	102.94 (3)	Nd2^i^—Si2—Nd3	140.17 (6)
Si2^i^—Nd2—Nd3^xi^	159.76 (3)	Nd3—Si2—Nd1^xii^	71.70 (3)
Si2—Nd2—Nd3^xi^	69.76 (3)	Nd3^ii^—Si2—Nd1^xii^	69.93 (3)
Si2—Nd2—Nd3^x^	54.35 (3)	Nd3—Si2—Nd3^x^	137.09 (5)
Si2—Nd2—Si1^xii^	95.25 (4)	Nd3—Si2—Nd3^ii^	78.55 (4)
Si2—Nd2—Si1^i^	140.41 (4)	Nd3^ii^—Si2—Nd3^x^	136.04 (5)
Si2^i^—Nd2—Si1^i^	47.67 (4)	Si1—Si2—Nd1^xii^	120.80 (6)
Si2^x^—Nd2—Si1^xii^	140.61 (4)	Si1—Si2—Nd2^i^	66.76 (5)
Si2^x^—Nd2—Si1^i^	97.55 (4)	Si1—Si2—Nd2^xiii^	67.28 (5)
Si2^x^—Nd2—Si1^x^	48.93 (4)	Si1—Si2—Nd2	135.73 (6)
Si2—Nd2—Si1^x^	124.91 (4)	Si1—Si2—Nd3^ii^	71.20 (5)
Si2—Nd2—Si2^x^	100.44 (2)	Si1—Si2—Nd3	138.40 (7)
Si2—Nd2—Si2^i^	93.45 (5)	Si1—Si2—Nd3^x^	64.85 (5)

Symmetry codes: (i) −*y*+1, −*x*+1, −*z*+1/2; (ii) −*y*+3/2, *x*+1/2, *z*+1/4; (iii) *x*+1/2, −*y*+3/2, −*z*+3/4; (iv) −*x*+1, −*y*+1, *z*+1/2; (v) −*x*+1, −*y*+2, *z*+1/2; (vi) −*y*+2, −*x*+1, −*z*+1/2; (vii) −*y*+3/2, *x*−1/2, *z*+1/4; (viii) *y*, *x*, −*z*+1; (ix) *x*−1/2, −*y*+3/2, −*z*+3/4; (x) −*x*+3/2, *y*−1/2, −*z*+1/4; (xi) *y*, *x*, −*z*; (xii) *y*−1/2, −*x*+3/2, *z*−1/4; (xiii) −*x*+3/2, *y*+1/2, −*z*+1/4; (xiv) −*y*+1, −*x*+2, −*z*+1/2; (xv) −*x*+1, −*y*+1, *z*−1/2; (xvi) −*x*+1, −*y*+2, *z*−1/2; (xvii) *y*+1/2, −*x*+3/2, *z*−1/4.
